# A New Device to Automate the Monitoring of Critical Patients' Urine Output

**DOI:** 10.1155/2014/587593

**Published:** 2014-01-28

**Authors:** Abraham Otero, Andrey Apalkov, Roemi Fernández, Manuel Armada

**Affiliations:** ^1^Department of Information and Communications Systems Engineering, University San Pablo CEU, 28668 Madrid, Spain; ^2^Centre for Automation and Robotics CSIC-UPM, Carretera Campo Real Km 0,200, La Poveda, Arganda del Rey, 28500 Madrid, Spain

## Abstract

Urine output (UO) is usually measured manually each hour in acutely ill patients. This task consumes a substantial amount of time. Furthermore, in the literature there is evidence that more frequent (minute-by-minute) UO measurement could impact clinical decision making and improve patient outcomes. However, it is not feasible to manually take minute-by-minute UO measurements. A device capable of automatically monitoring UO could save precious time of the healthcare staff and improve patient outcomes through a more precise and continuous monitoring of this parameter. This paper presents a device capable of automatically monitoring UO. It provides minute by minute measures and it can generate alarms that warn of deviations from therapeutic goals. It uses a capacitive sensor for the measurement of the UO collected within a rigid container. When the container is full, it automatically empties without requiring any internal or external power supply or any intervention by the nursing staff. In vitro tests have been conducted to verify the proper operation and accuracy in the measures of the device. These tests confirm the viability of the device to automate the monitoring of UO.

## 1. Introduction

Critical care unit staff has the support of multiple monitoring devices capable of measuring most of the patient's physiological parameters. More often than not, these devices also check whether these parameters remain within acceptable values, and they alert the healthcare staff (usually via audible warnings) when the parameters take values that pose a risk to the patient's life [1, 2]. Patient monitoring devices reduce the workload of the healthcare staff, since they need not continuously check the values of the physiological parameters of every patient [3, 4]. A physiological parameter that is still measured manually and therefore has not benefited from the automation of monitoring is urine output (UO). This parameter is the best indicator of the state of the patient's kidneys. When the kidneys are producing an adequate amount of urine it means that they are well perfused and oxygenated. Otherwise, it indicates that the patient is suffering from some pathology. UO is used in multiple therapeutic protocols to assess how the patient reacts to treatment, such as the resuscitation of septic shock patients [5, 6] and the resuscitation and early management of burn patients [7, 8].

Critical patients' urine is collected in a graduated container which is often divided into several chambers with an overall capacity of approximately 500 mL. This container is connected to a 1500–3000 mL plastic bag. Every hour, the reading of the container of every patient must be manually recorded. This requires walking to the patient's bed, taking the measure of UO production visually, writing it down on the nursing documentation sheet, opening the valve that releases urine from the graduated container to the plastic bag, waiting for the urine to drain, closing the valve, checking that the valve is properly sealed, and checking if the plastic bag needs to be emptied. This entire process can take up to two minutes [9]. In a critical care unit with 10 patients, this means 20 minutes per hour, 8 hours a day. The automation of some steps of this process, or ideally of all the steps, could save a considerable amount of work. Furthermore, the frequency of measuring UO is determined not by physiological reasons, but by the convenience of its measurement over rather long periods of time such as one hour. A more continuous measurement of UO could permit the identification of changes in UO at earlier stages, with the associated potential for improving patient outcomes [10–14]. There is already preliminary evidence pointing to this direction [15–18].

This paper presents a patent pending device [19] that completely automates most of the tasks related to the monitoring of the UO of critical patients. This device extends a solution previously developed by the authors to automate the measurements of UO [20] by automating the emptying of the container where urine collects, without requiring any internal or external power supply or actuators.

## 2. Materials and Methods

**Figure 1:**
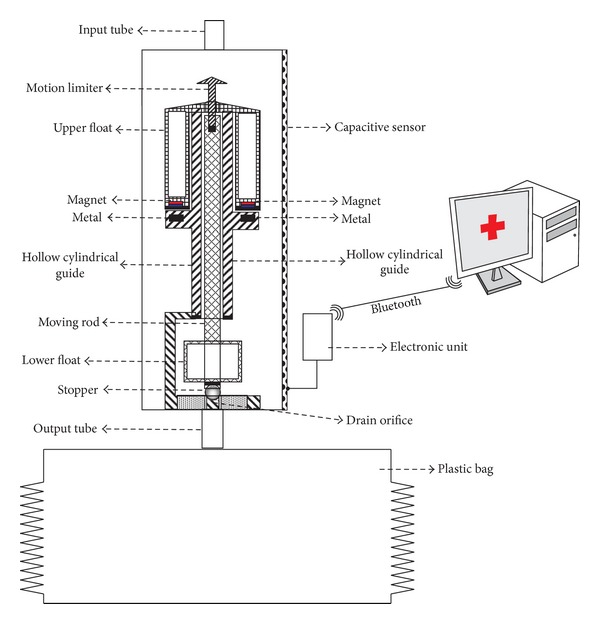
Device design.

Our device has a container with a single 90 mL chamber. This container receives the urine through a flexible tube, which in turn is connected to a Foley catheter. In its outer wall it has a capacitive sensor (see Figure 1). The longitudinal blade of the capacitive sensor must be at least as long as the container's height to provide a correct measurement of the height of the column of liquid (urine) inside the container. An evaluation of the use of capacitive sensors for measuring the amount of urine within a rigid container can be found in [20]; when used to measure minute-by-minute UO the error of these sensors is below 5%.

When full, the container that collects the urine will be emptied automatically without requiring electrical power. To achieve this, the device uses magnetic forces to prevent the activation of the emptying mechanism of the container until it is nearly full of urine. The drain orifice is closed by a stopper placed at the end of a moving rod to which two floats are coupled. In the absence of magnetic force, a small amount of liquid in the container would be sufficient for the floats to pull up the rod and the stopper and therefore to trigger the emptying. But the magnetic force prevents this from happening until the container is almost full; only at this point does the buoyancy force overcome the magnetic force. Then the magnetic force disappears because the magnet moves away from the metal surface. Before the magnetic force can reappear, most of the liquid in the container has to be drained.

**Figure 2:**
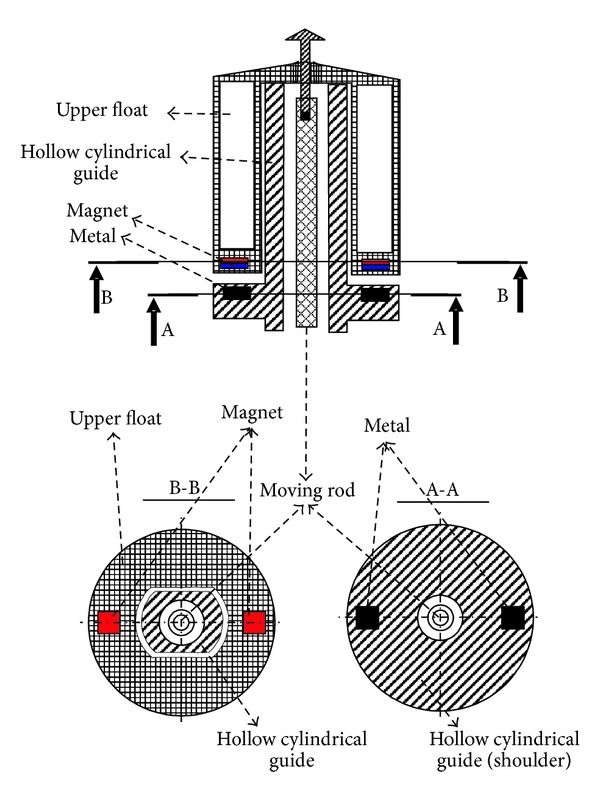
Location of the magnets and metal pieces in the upper float and on the shoulder of the hollow cylindrical guide.

We shall describe now the balance of the different forces that come into play at different points during filling. When the container is empty the exit hole is sealed by the stopper that is on the end of a rod. The rod has a float located in its lower part and a motion limiter at the top (see Figure 1). The rod passes through the interior of a hollow cylindrical guide attached to the base of the container. Let *W* be the sum of the weights of the rod, the lower float, the stopper, and the motion limiter. Note that the weight of all these elements is falling over the stopper. If there is no liquid inside the container, this is the only force acting on the stopper. There is a second float surrounding the top portion of the cylindrical guide that rests on a shoulder of the cylindrical guide. At the bottom of the upper float there are two magnets located at opposite sides of the diameter of the float (see Figure 2). In the shoulder of the cylindrical guide there is a piece of metal. If the second float rises, the rod's motion limiter will come into contact with this float.

**Figure 3:**
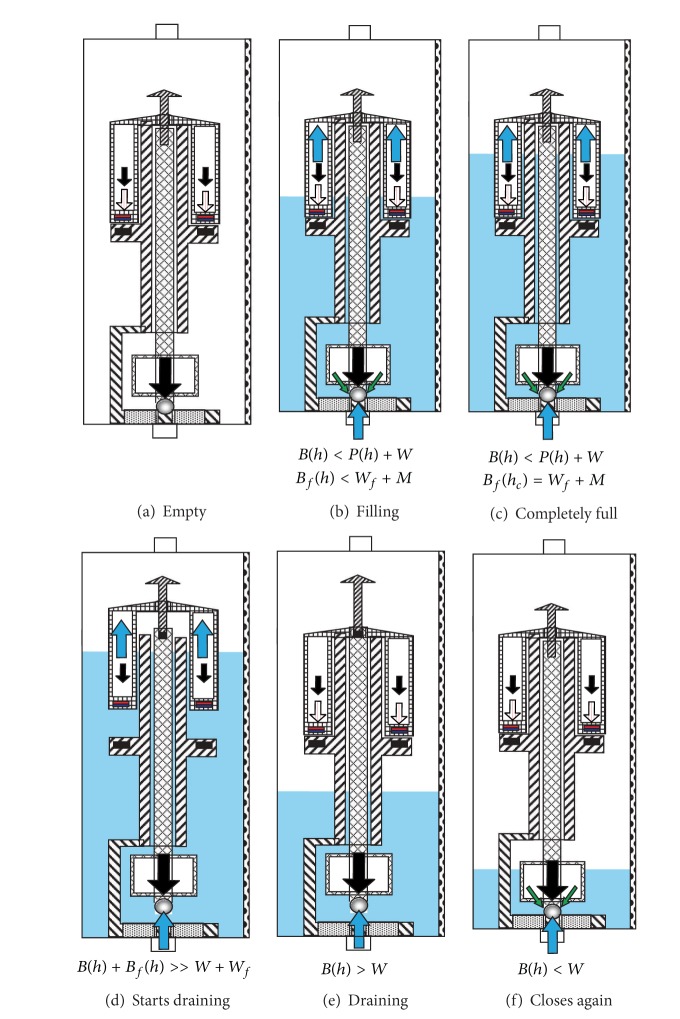
Functioning of the device. Each subfigure shows the forces that come into play in each of the states. The solid black downwards pointing arrows correspond to forces caused by gravity; the solid blue upwards pointing arrows to buoyancy forces; the dashed red downwards pointing arrows to magnetic forces; and the green inclined arrows to forces caused by the pressure of the column of liquid.

When the container starts filling, two new forces come into play. There is the buoyancy force that equals the weight of the volume of liquid displaced by the rod, the lower float, and the stopper (see Figure 3(b)). We shall call the buoyancy force *B*(*h*), where the (*h*) indicates that it depends on the height of the column of liquid. The second force is the one caused by the pressure of the column of liquid on the stopper. We shall call this force, which also depends on the height of the column of liquid, *P*(*h*) (see Figure 3(b)). The rod, the lower float, the stopper, and the motion limiter are designed so that for any height of the column of liquid the buoyancy force (which pulls up the stopper) is lower than the pressure of the column of liquid on the stopper plus the weight of these components. Therefore, in the absence of any other forces, the stopper never opens, regardless of the height of the column of liquid.

When the liquid reaches the second float, three different forces are acting on it: the weight of the float (*W*_*f*_), the buoyancy force that depends on the height of the column of liquid (*B*_*f*_(*h*)), and the magnetic force that helps maintain the float on the shoulder of the cylindrical guide (*M*). Initially the weight of the float plus the magnetic force exceeds the buoyancy force (see Figure 3(b)). However, *W*_*f*_ and *M* are constant, while *B*_*f*_(*h*) increases with the height of the column of liquid. For some value *h*_*c*_ of the height of the column of liquid it holds that the buoyancy force is equal to the magnetic force plus the weight of the upper float (see Figure 3(c)). When more liquid enters the container, the buoyancy force will overcome the other two forces and the float will rise. The magnetic force *M* falls to zero and the float rises rapidly and hits the motion limiter, pulling up the rod and the stopper (see Figure 3(d)). At this point the force caused by the pressure of the column of liquid on the stopper also disappears. This permits most of the liquid from the container to be drained (see Figure 3(e)) before it holds that *B*(*h*) < *W* (see Figure 3(f)). At that time, the sum of the weights of the rod, the lower float, the stopper, and the motion limiter overcomes the buoyancy force, and the stopper closes the drain orifice. The container begins to be filled again, repeating the same cycle, although this time it starts with a small amount of liquid inside the container.

**Figure 4:**
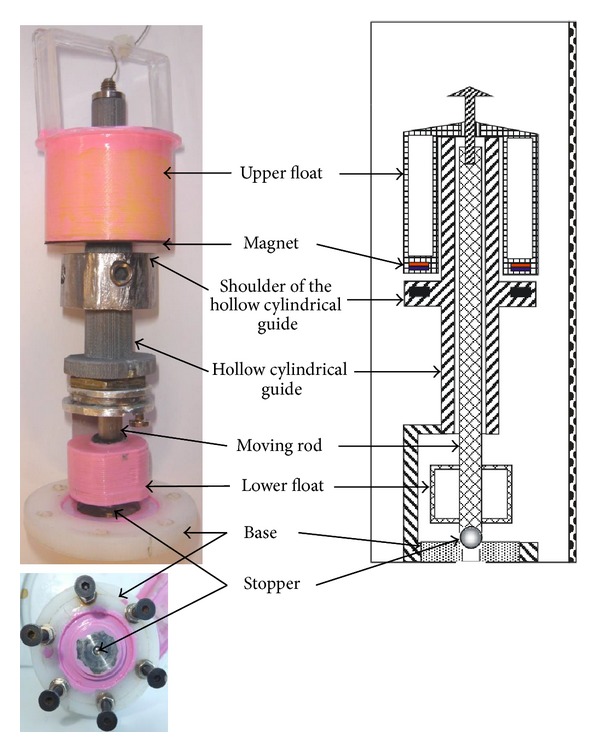
On the left we show lateral and bottom views of our prototype. On the right we show the corresponding parts in the design diagram.

In our prototype (see Figure 4) the total mass of the assembly formed by the rod, the stopper, the lower float, and the motion limiter is 2.6 g, while the upper float has a mass of 5.5 g. The force *W* (measured in Newtons) is 0.049 N. For the forces *P*(*h*) and *A*(*h*) it holds that 0 < *P*(*h*) < 0.0064 N and 0 < *A*(*h*)< 0.0549 N, depending on the height of the column of liquid inside the container. But for any height, it holds that *A*(*h*) < *P*(*h*) + *W*. When the container is filled and the upper float overcomes the magnetic force (that is approximately 0.09 N), it rises with a kinetic energy approximately equal to 0.1 N, thus opening the stopper.

The capacitive sensor we used was manufactured by Sensortechnics GmbH. An interface circuit was built to enable communication between the sensor and a serial port to Bluetooth adapter, which sends the readings to and receives the commands from the central PC (see Figure 1), although any other mechanisms of wired or wireless communication with the PC could have been used. The UO readings are acquired by a Java program running on a PC, which provides the health care staff with minute-by-minute measurement of the patient's UO and permits the triggering of alarms if this production deviates from the therapeutic goals.

### 2.1. Some Additional Considerations

For the correct operation of the stopper, it must have a highly polished smooth surface, and it must be made of a corrosion resistant material. The patient's urine may contain hard inorganic and soft organic sediments that may cause the stopper not to close properly. In order to avoid this, either the stopper or the walls of the hole, or both, must be made of a soft material such as silicone, so that the stopper can block the outflow of liquid even in the presence of sediments. To prevent the rod from adhering to the hollow guide, the rod should not have flat surfaces. It must have a conical or spherical surface. The top of the second float and the top of the motion limiter must not have a flat surface, since in this case fluid could accumulate on them and would change the balance of forces, which may cause the device to malfunction. To avoid this, the top of the second float and of the motion limiter must have a pyramidal or conical shape.

### 2.2. In Vitro Testing Setup

**Figure 5:**
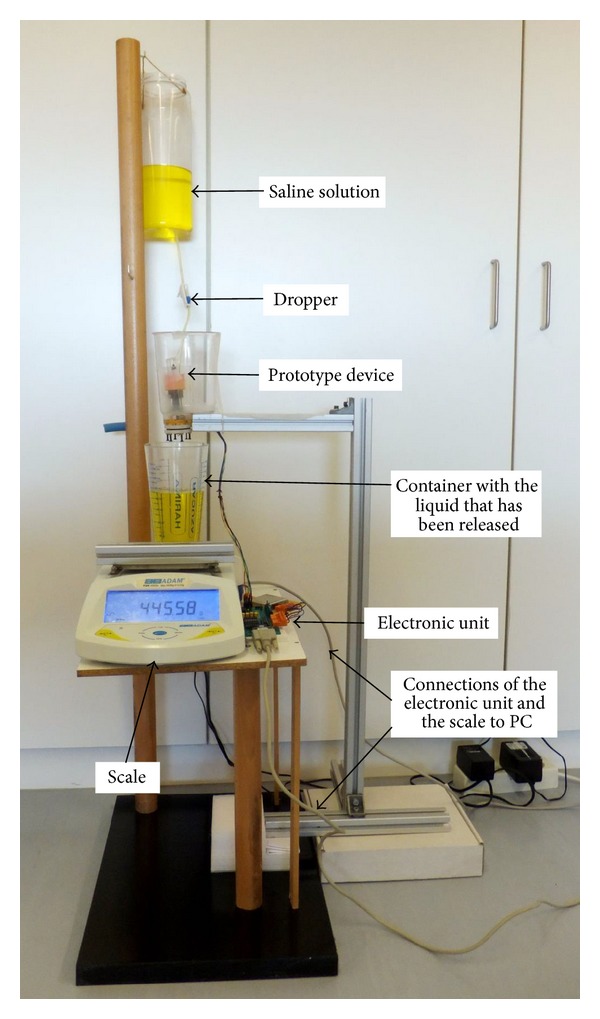
Setup used in the validation of the device.

To verify the proper operation of the prototype we have used a saline solution with similar properties to urine. A dropper was used to simulate the urine flow into the device (see Figure 5). The prototype was positioned so that through automatic release the liquid inside the rigid container would fall into a container located on the plate of a high-precision industrial scale, a PGW 4502e, built by Adam Equipment Inc. This scale is used to determine if the liquid released from our device is released at the right level of filling of the container and if the right amount of fluid is released before the stopper closes the drain orifice. The scale is equipped with a serial port that permits querying for readings. We built a program that periodically takes measurements from the scale. This program was running on a PC which was connected with the scale through the serial port. We can determine the volume of liquid in the container located on the plate of the scale at any time by subtracting the weight of the empty container from the weight measured by the scale and dividing the result by the saline solution density; that is, from the scale's measures we can calculate the amount of liquid that has been released each time.

This setup permits the automation of the process of carrying out multiple measures of the fluid drained by the device each time its content is released. Given that the PGW 4502e scale has an accuracy guaranteed by the manufacturer of 0.01 g, we shall consider that measures obtained from the scale are the ground truth which we shall use to determine how reliable the device is; that is, the release of fluid from the device always occurs when the liquid reaches the same level and always drains the same amount of fluid before starting to accumulate liquid again.

## 3. Results and Discussion

Using the in vitro testing setup described in Section 2.2, we measured a total of 50 discharges of the prototype, grouped in 10 experiments of 5 discharges in each. In the experiments, three different rates of urine production were simulated using the dropper. The urine production rates corresponded approximately to 500, 1500, and 3000 mL/day. 10 discharges were performed at 500 mL/day, while 20 discharges were performed at 1500 and 3000 mL/day. The device released 88.1 ± 1.4 mL, 89.2 ± 1.7 mL, and 88.3 ± 1.8 mL at 500, 1500, and 3000 mL/day, respectively. According to the theoretical calculations based on the dimensions and geometry of the device, it should release 90 mL in each discharge.

### 3.1. Discussion

Automating the monitoring of UO can provide the same benefits that the automation of the monitoring of many other physiological parameters has already brought to critical care units: decreasing the workload of the health care staff, simplifying the construction of digital records of the patient, and providing more frequent measures of the parameter. As we have already argued, in a critical care unit with 10 patients, up to 8 hours of health staff time a day are used in tasks related to the monitoring of UO. If these tasks were automated, there may be an improvement in patient outcomes equivalent to an increase in the staffing of the unit proportional to the saved time [21–25]. The device we have presented in this paper automates all tasks related to the monitoring of UO, except the changing of the plastic bag where urine accumulates. We estimate that this task in a critical care unit with 10 patients requires about 60 minutes a day. Therefore, the device presented here would save up to 7 hours of work per day per 10 patients.

There are a few papers in the scientific literature describing solutions to automate some of the steps involved in the UO monitoring process. At the end of the 1980s several automatic urine meters based on ultrasound sensors were proposed [26, 27]. The low accuracy of this type of sensor makes it unsuitable to measure minute-by-minute UO. These products were discontinued and they are not marketed today. Laser sensors have been proposed for use in the measure of the amount of liquid contained in a recipient with more accuracy than ultrasound sensors. However, their use is rather cumbersome because when a laser beam hits a liquid surface most of the beam power is transmitted. Float devices equipped with mirrors have been proposed to address this problem [28], but the final cost of the sensor makes it prohibitive for a device (the urine container) that must be disposable.

Hersch et al. developed a device capable of measuring urine output every minute using a photoelectric cell that counts drops of urine [9]. Solutions for this problem have also been proposed using high precision scales [29]. These solutions still require an hourly visit to the patient's bed to activate a valve to release the urine that it is collected in some container. In [30] a device capable of emptying itself automatically was presented; it used reed switches to measure UO. However, this solution has a number of drawbacks that prevent it from being taken to the clinical routine [20], and it cannot provide minute-by-minute measures. A device based on capacitive sensors to provide minute-by-minute measures of urine output was proposed in [20]. Compared with this device, the one introduced in this paper has the advantage of requiring a single capacitive sensor, thereby reducing the manufacturing cost, and of automatically emptying the container that collects the urine, avoiding the hourly bedside visit.

A disadvantage of the device presented in this paper when compared with the manual urine meters currently used in critical care units is its use of metals and magnets. Manual urine meters can be made entirely of plastic and therefore are MRI compatible. Our device should be disconnected from the patient before performing an MRI.

## 4. Conclusion

We have designed and built a device capable of automatically monitoring the UO of critical care patients. This device automates all tasks related to the monitoring of UO, with the exception of emptying the plastic bag that collects the urine. Currently this parameter is measured and monitored manually by nursing staff, which requires at least one supervisory visit to the patient's bedside every hour. We estimate that this device could save up to 7 hours of nursing staff work per day per 10 patients. Furthermore, with this device more frequent urine production measures can be taken (up to one per minute). In the literature there is evidence that indicates that more frequent UO measurement can impact clinical decision making and improve patient outcomes [15–18] and hence the interest of the device presented in this paper.
